# Multi-omics analysis reveals the mechanism of *Lactobacillus plantarum* in alleviating metabolic disorders in type 2 diabetic mice through the gut-liver axis

**DOI:** 10.1128/msystems.00965-25

**Published:** 2025-10-30

**Authors:** Caili Zhang, Yue Gao, Yixuan Huang, Yunyi Qin, Yongxin Li, Qingru Chen, Tiantian Wu, Yujie Zhang, Yan Zhang, Datong Deng, Binbin Huang, Mingwei Chen, Maozhen Han

**Affiliations:** 1 School of Life Sciences, Anhui Medical University 428675 , Hefei, Anhui, China; 2 Microbial medicinal resources development research team, Anhui Provincial Institute of Translational Medicine, Hefei, Anhui, China; 3 Department of Clinical Medicine, The First School of Clinical Medicine, Anhui Medical University 12485, Hefei, Anhui, China; 4 School of Life Sciences, Hefei Normal University 177524, Hefei, Anhui, China; 5 School of Public Health, Anhui Medical University 569061, Hefei, Anhui, China; Institut de Recherche pour le Developpement Delegation Regionale Occitanie, Montpellier, France

**Keywords:** gut microbiota, *Lactobacillus plantarum*, T2DM, multi-omics

## Abstract

Type 2 diabetes mellitus (T2DM) mainly induced by high-fat diets (HFD) is becoming a prevalent and serious metabolic disease worldwide. Although probiotics are considered a treatment for T2DM, the underlying mechanisms and therapeutic effects of the specific strain remain unclear. Thus, elucidating the strain-dependent mechanisms and identifying effective probiotic strains for T2DM management are critically needed. In this study, *Lactobacillus plantarum* was administered to HFD-induced T2DM mice to evaluate its effects on fasting blood glucose (FBG), body weight (BW), gut microbiota (GM) composition, gut metabolites, and liver gene expression. We found that *L. plantarum* supplementation significantly reduced FBG, insulin levels, and BW, while ameliorating liver and pancreatic damage in T2DM mice. Furthermore, shifts in gut microbial composition were observed following *L. plantarum* intervention, characterized by the suppression of *Lactobacillus johnsonii*, *Bacteroides acidifaciens*, *Alistipes* sp., and bacterium D1676, along with enrichment of beneficial strains, such as *L. acidophilus*, *Enterorhabdus* sp. P55, and *Bacteroides caecimuris*. Besides, *L. plantarum* administration significantly increased the concentrations of glycocholic acid, arachidonic acid, L-tryptophan, and palmitic acid while decreasing chenodeoxycholic acid concentration. This modulation is accompanied by the upregulation of *Hmgcr* and *Ugt1a5* mRNA expression, leading to increased levels of *TGR5* (G protein-coupled bile receptor) and *GLP1R* (Glucagon-like peptide-1 receptor). Transcriptomic and metabolomic analyses confirmed that *L. plantarum* alleviates T2DM caused by HFD through upregulating the bile acid secretion pathway. The results demonstrate that *L. plantarum* ameliorates symptoms in T2DM mice through the gut-liver axis.

**IMPORTANCE:**

T2DM is becoming a global health problem linked to poor diet and metabolic disorders. The probiotic *L. plantarum* offers a promising natural approach by targeting two key factors: gut bacteria balance and bile acid function. In HFD-fed mice, this treatment helps restore healthy gut microbes and improves bile acid signaling, which together lower blood glucose, reduce inflammation, and protect against liver damage. Unlike many diabetes medications, *L. plantarum* works with the body’s natural systems, potentially providing a safer, long-term solution. This research highlights how probiotics could complement existing therapies, offering novel therapeutic strategies for diabetes treatment through the gut-liver axis.

## INTRODUCTION

Obesity is a persistent metabolic disorder characterized by abnormal fat accumulation, which markedly increases the illness risk of developing multiple health complications, such as T2DM (1). Presently, roughly 537 million people globally suffer from diabetes, with projections indicating a rise to 783 million by 2045, with T2DM making up a staggering 90% (2). T2DM has become a growing worldwide health concern (3). The pathological mechanism of T2DM is intricate, involving a multitude of factors that interact with each other, such as genetic factors (4), environmental factors (5), gut microbes (6), and dietetic factors (7). There is a notable correlation between changes in diet and the variations in gut microbiota community structure (8, 9). The taxonomical and functional compositions of gut microbial communities are increasingly acknowledged as vital to glucose regulation and insulin responsiveness (10). Mounting evidence indicates that the dysbiosis of gut microbiota is extremely associated with the pathogenesis of T2DM (11–13). Therefore, our present study focuses on T2DM and investigates therapeutic strategies through perspectives of gut microbiota modulation, probiotic supplementation, and integrated multi-omics data analysis.

In recent decades, research has shown that the activity of gut microbiota and the substances they produce are crucial for keeping our metabolic processes running smoothly. Changes in these microbial communities are closely linked to various metabolic health issues (14, 15). Recent studies highlight several probiotics, such as *Akkermansia muciniphila* (16), *Lactobacillus casei* (17), *Bifidobacterium animalis* (18), and *Lactobacillus plantarum* (19), which have the potential ability in managing diabetes by promoting gut microbiome equilibrium (20). *L. plantarum* has been shown to ease inflammation, regulate the body’s metabolism, and combat obesity. Research indicates that *L. plantarum* can restore harmony within the intestinal microbial community and impact the host’s metabolic health by generating short-chain fatty acids (21–23). Regular intake of *L. plantarum* can lead to measurable reductions in both FBG and postprandial blood glucose (PPG) levels among T2DM individuals. While the extent of improvement varies across studies, this beneficial effect has been consistently observed. At the same time, the inhibitory effect of *L. plantarum* on inflammatory factors helps improve the chronic inflammatory state associated with T2DM (24, 25), laying a foundation for its application in diabetes management.

However, although a series of experiments and work have been conducted to explore the function and mechanisms of *L. plantarum* on T2DM and other metabolic diseases, its underlying mechanisms still require further investigation. For example, previous studies have demonstrated that *L. plantarum* reduces both FBG and glycated hemoglobin (HbA1c) levels (26), but the underlying mechanisms remain unexplored and unclear. Additionally, in prior studies exploring the mechanisms of *L. plantarum* in metabolic diseases, including T2DM, the approach was primarily focused on single aspects, such as gut modulation by *L. plantarum* or its anti-inflammatory effects (27), This focus yields only a partial understanding of the mechanism and limits its clinical application. Therefore, to deepen the understanding of the mechanisms of *L. plantarum* on T2DM and to broaden knowledge of microbiome-based approaches for the clinical treatment of T2DM, a combination of animal experiment and multi-omics approaches should be applied, such as the integrated usage of metagenomics sequencing, untargeted metabolomics, and transcriptomics (28, 29).

In this present study, we explored how *L. plantarum* might alleviate T2DM mice by targeting the gut microbiome. Using a combination of animal models and multi-omics analysis, we delved into the underlying mechanisms of probiotic therapy, paying close attention to shifts in gut microbiota composition, their metabolic byproducts, and how these changes influence hepatic gene expression. HFD-induced T2DM mice were administered *L. plantarum* [10^7^ CFU/(g*day)] by oral gavage for 12 weeks. Their metabolic parameters were then compared with those of healthy (NC + SPSS) and diabetic (HFD + SPSS) control groups that received stroke-physiological saline solution (SPSS). Throughout the course of treatment, we tracked changes in body weight and blood glucose levels of mice to assess the intervention effectiveness of *L. plantarum*. During the experimental period, fecal samples were collected at two key time points: upon successful model establishment (week 12) and following treatment completion (week 24). These samples were analyzed using metagenomic sequencing and liquid chromatograph-mass spectrometer (LC-MS) to evaluate changes in the gut microbiota and related metabolites, respectively. Additionally, we weighed pancreatic, hepatic, and adipose tissues, which were then subjected to hematoxylin-eosin (H&E) staining. Liver tissues underwent transcriptomic analysis to examine molecular changes induced by *L. plantarum*. The findings revealed that *L. plantarum* significantly curtailed weight gain and lowered fasting blood glucose levels in the diabetic mice. Transcriptomic data further indicated that *L. plantarum* helps mitigate liver fat accumulation by upregulating several genes, such as *Fasn* (fatty acid synthase) and *Cpt1a* (carnitine palmitoyltransferase 1A). An integrated analysis of transcriptomic and metabolomic data sets uncovered that differentially expressed genes (DEGs) and differential metabolites (DMs) were co-enriched in the bile acid secretion pathway, with DEGs (e.g., *Hmgcr*, *Ugt1a5*) and DMs (e.g., chenodeoxycholic acid, glycodeoxycholic acid) exhibiting coordinated regulation (*r* > 0.5, *P* < 0.05). This multi-omics convergence highlights bile acid metabolism as a central axis through which *L. plantarum* modulates host–microbiota crosstalk in T2DM.

In conclusion, our findings reveal that *L. plantarum* modulates microbial composition, regulates metabolic pathways, and alters liver gene expression networks associated with lipid metabolism and energy homeostasis. This comprehensive investigation elucidates the anti-obesity mechanisms of *L. plantarum* and offers scientific evidence for its possible use in treating metabolic conditions.

## MATERIALS AND METHODS

### *L. plantarum* preparation

Fermented sauerkraut samples were aseptically collected during the mid-fermentation phase (OD₆₀₀: 0.4–0.6) to maximize lactic acid bacterial diversity. Approximately 1 mL of fermentation fluid was transferred into sterile tubes, followed by serial 10-fold dilutions in SPSS. Diluted samples were inoculated into de Man, Rogosa, and Sharpe (MRS) broth and cultured anaerobically at 37°C for 18–24 h. Bacterial suspensions were plated on MRS agar and incubated anaerobically at 37°C for 48–72 h. Colonies exhibiting typical *Lactobacillus* morphology (creamy-white, convex, 1–3 mm diameter) were purified. The 16S rRNA gene was amplified using universal primers 27F (5′-AGAGTTTGATCCTGGCTCAG-3′) and 1492R (5′-GGTTACCTTGTTACGACTT-3′). Purified PCR products were bidirectionally sequenced, and the resulting sequences were compared to the NCBI RefSeq rRNA database via BLASTn. A neighbor-joining phylogenetic tree (bootstrap = 1,000) constructed in MEGA 11 confirmed the strain as *L. plantarum* (Fig. S1). The bacterial suspension was adjusted to 1 × 10⁹ CFU/mL in 0.9% SPSS (30).

### Experimental design of animals

**Fig 1:**
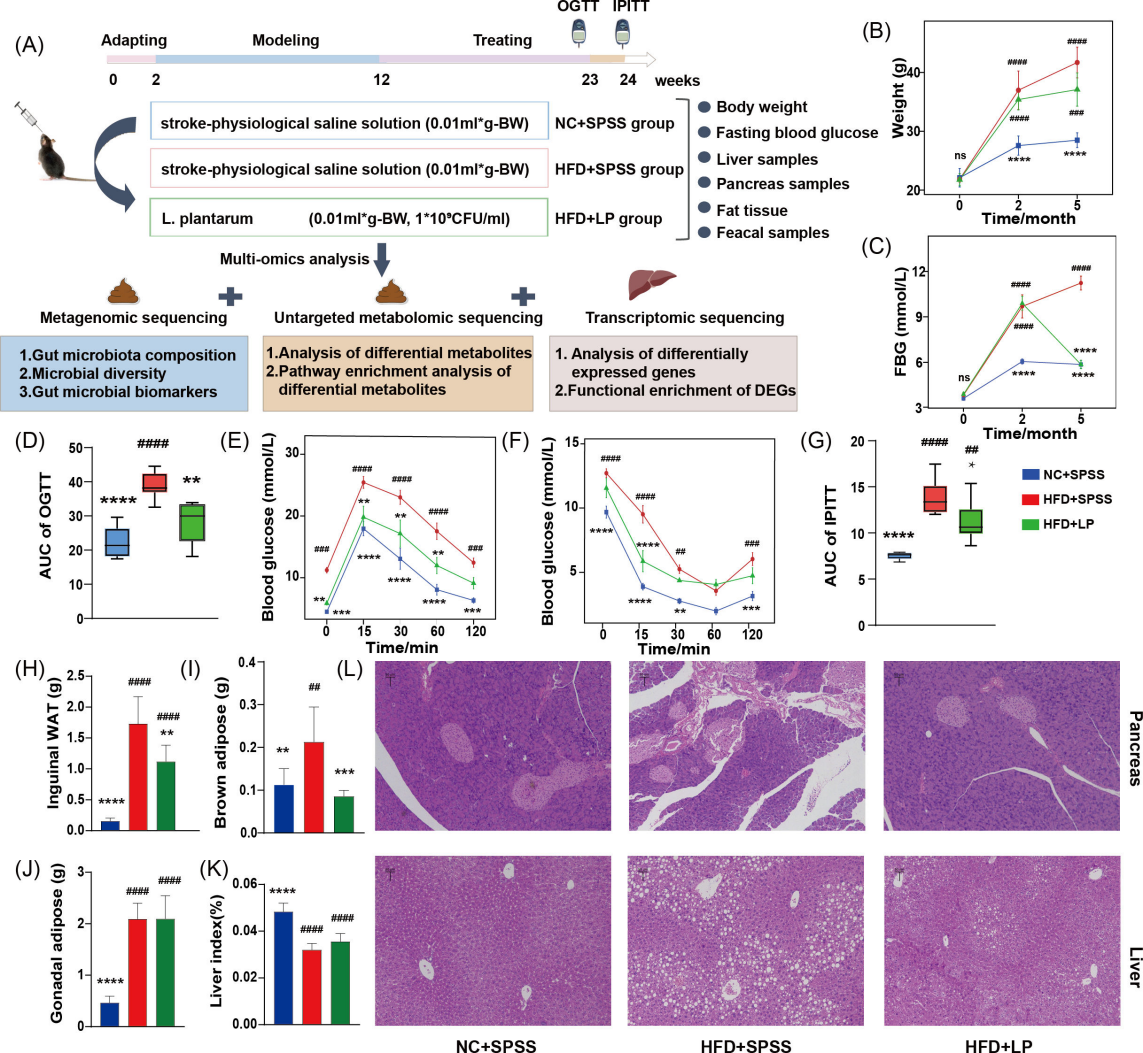
Oral administration of *L. plantarum* to T2DM mice resulted in significant reductions in BW and FBG levels, while also ameliorating pancreatic and splenic damage. (**A**) Experimental design and procedures for studying the potential mechanisms of *L. plantarum* intervention in HFD-induced T2DM. (**B**) Body weight data were collected from all groups at three phases: pre-modeling (2 weeks), post-modeling (12 weeks), and post-intervention (24 weeks). (**C**) The differences in FBG levels among experimental groups were characterized throughout the study period. (**D**) OGTT analysis revealed significantly reduced AUC values in both NC + SPSS and HFD + LP group mice compared with the HFD + SPSS group. (**E**) Blood glucose concentrations were assessed in the NC + SPSS, HFD + SPSS, and HFD + LP group following glucose ingestion (1.5 g/kg). (**F**) We monitored blood glucose concentrations at 0, 15, 30, 60, and 120 min after intraperitoneal insulin injection (0.75 U/kg) across all experimental groups. (**G**) IPITT revealed that the AUC for NC + SPSS and HFD + LP groups was notably less than that of the HFD + SPSS group. (**H**) inguinal WAT, (**I**) brown adipose, (**J**) gonadal adipose tissue, and (**K**) liver indices were measured across all experimental groups mice to compare the effects of *L. plantarum* on T2DM mice. (**L**) Pancreas and liver sections were analyzed by H&E staining. ####, *P* < 0.0001; ###, *P* < 0.001; ##, *P* < 0.01; #, *P* < 0.05 (compared with NC + SPSS group). ****, *P* < 0.0001; ***, *P* < 0.001; **, *P* < 0.01; *, *P* < 0.05 (compared with HFD + SPSS group).

A total of thirty healthy male C57BL/6J mice (six weeks old, 20–25 g) were obtained from Liaoning Changsheng Biotechnology Co., Ltd. These specific pathogen-free (SPF) animals were housed under controlled sterile conditions maintained at 22 ± 1°C and 45–55% relative humidity. After being fed a standard chow diet for 2 weeks, mice were randomly assigned to two groups via simple randomization: a high-fat diet (HFD, *n* = 20) group and a normal chow control group (NC, *n* = 10). Each cage contained five mice with unrestricted access to food and water. The HFD group received a high-fat diet, while the NC group was fed a standard diet and SPSS (NC + SPSS). After 10 weeks of high-fat diet feeding, FBG levels were measured following a 12-hour overnight fast, with hyperglycemia defined as FBG >9.5 mmol/L (31). The T2DM mouse model was successfully constructed. The HFD group was randomly split into two equal groups of 10 mice each. One group received SPSS via oral administration (HFD + SPSS), while the other was treated with *L. plantarum* (HFD + LP). Both groups underwent a 12-week intervention period under identical experimental conditions. Twelve weeks after treatment, we collected fecal samples from all groups and measured their BW and FBG. After the experiment, mice were anesthetized with urethane. Following orbital blood collection, the mice were euthanized by cervical dislocation. Then, the mice liver, gonadal adipose tissue (GAT), inguinal white adipose tissue (inguinal WAT), and brown adipose tissue (BAT) were isolated and weighed (Fig. 1A).

### H&E staining, oral glucose tolerance tests (OGTT), intraperitoneal glucose tolerance tests (IPITT), and multi-omics analysis

The staining procedure followed the earlier research (32). Pancreatic and hepatic tissues were processed for histology (paraformaldehyde-fixed, paraffin-embedded, H&E-stained) and examined by light microscopy. After 23 weeks, mice underwent an oral glucose tolerance test (1.5 g/kg) with blood glucose measured at 0, 15, 30, 60, and 120 min to calculate AUC. At week 24, an IPITT test was conducted to assess insulin sensitivity. Mice were fasted for 12 h (with water) and injected intraperitoneally with insulin (0.75 U/kg). Tail vein blood glucose levels were measured at 0, 15, 30, 60, and 120 min post-injection using a glucose meter. Fecal samples were analyzed using metagenomics and metabolomics, and liver samples were subjected to transcriptomic sequencing. Detailed experimental procedures and bioinformatics analysis were provided in the Supplemental material.

### Statistical analysis

GraphPad Prism (v9.5.1) was used for statistical analyses, all data were presented as mean ± standard error mean (SEM), and *P* < 0.05 was considered statistically significant (*P < 0.05; **P < 0.01; ***P < 0.001; ****P < 0.0001). Two-tailed Student’s *t*-test (for parametric data) or a Mann–Whitney *U* test (for non-parametric data) was used to evaluate the significance of differences between the two groups. When analyzing more than two groups, a one-way ANOVA was used to gage significance, followed by either Tukey’s or Dunnett’s *post hoc* tests (for parametric data) or the Kruskal–Wallis test with Dunn’s method for multiple comparisons (for non-parametric data).

## RESULTS

### *L. plantarum* can decrease the level of FBG and BW in T2DM mice

We established an HFD-induced diabetic mice model to estimate the hypoglycemic effects of *L. plantarum*. At the end of the modeling period, the BW of the HFD + SPSS group was significantly higher than that of the NC + SPSS group (NC + SPSS: 27.57 ± 1.63 g, HFD + SPSS: 36.99 ± 3.26 g, *P* < 0.0001, ANOVA; Fig. 1B). Following 12 weeks of *L. plantarum* intervention, in comparison with the HFD + SPSS group, the FBG levels in the HFD + LP group were significantly lower than those in the HFD + SPSS group (HFD + SPSS: 11.23 ± 1.2 mmol/L, HFD + LP: 5.87 ± 0.69 mmol/L, NC + SPSS: 5.87 ± 0.67 mmol/L, *P* < 0.0001, ANOVA; Fig. 1C).

For the OGTT, key parameters such as blood glucose levels at various time points and the area under the curve (AUC) are crucial for evaluating glucose tolerance in T2DM mice. AUC values in the HFD + LP group (28.22 ± 2.54) were elevated versus NC + SPSS group (22.24 ± 1.85), but decreased compared to the HFD + SPSS (38.91 ± 1.51) group (*P* < 0.01, ANOVA; Fig. 1D). Specifically, the *L. plantarum*-treated mice displayed significantly lower blood glucose levels than the HFD + SPSS group at all time points, except 120 min post-glucose administration (*P* < 0.01, ANOVA; Fig. 1E), indicating improved glucose tolerance. The IPITT revealed significantly higher blood glucose levels in HFD + SPSS mice compared to NC + SPSS controls throughout the 120-minute period (*P* < 0.01). Notably, *L. plantarum* treatment effectively ameliorated hyperglycemic state (Fig. 1F). The results showed that the insulin sensitivity in the HFD + LP group was significantly higher than that of the HFD + SPSS group, but still lower than in the NC + SPSS group, with all differences being statistically significant (*P* < 0.05, ANOVA; Fig. 1G).

Regarding adipose tissue distribution, marked differences were observed among experimental groups. Compared to the NC + SPSS group, groin fat (0.15 ± 0.05 g) and brown fat (0.11 ± 0.04 g), HFD-fed mice exhibited substantial increases in both groin fat mass (1.73 ± 0.44 g) and brown adipose tissue weight (0.21 ± 0.08 g). The HFD + SPSS group has increased 1053% and 91% compared with the NC + SPSS group. Notably, treatment with *L. plantarum* effectively mitigated the adiposity changes induced by the HFD. Specifically, the HFD + LP group exhibited significant reductions in groin fat mass (1.12 ± 0.26 g) and brown adipose tissue (BAT) mass (0.09 ± 0.01 g) compared with the HFD + SPSS group (*P* < 0.01 by one-way ANOVA; Fig. 1H and I). Compared to the HFD + SPSS group, the HFD + LP group exhibited a 35% reduction in inguinal WAT (*P* < 0.01) and a 57% decrease in BAT weight (*P* < 0.001). However, no significant intergroup differences were detected in GAT mass (Fig. 1J). The hepatic index data revealed a marked decline in both the HFD + SPSS and HFD + LP groups compared to the NC + SPSS group. Interestingly, the HFD + LP group showed a slight uptick in liver index values relative to the HFD + SPSS group (Fig. 1K). These results demonstrate that oral administration of *L. plantarum* restored glycemic control in T2DM mice, attenuating both BW gain and FBG levels.

### Histopathological analysis reveals that *L. plantarum* intervention can ameliorate pancreatic and liver damage in T2DM mice

H&E staining revealed disorganized hepatocytes with rounded shapes and widespread cytoplasmic vacuolation in diabetic mice, indicating severe liver injury, which was notably mitigated in *L. plantarum*-treated mice. In pancreatic sections, the NC + SPSS group showed well-preserved architecture, while the HFD + SPSS group exhibited marked villous atrophy, rupture, and severe submucosal thickening. The HFD + LP group showed restored tissue structure compared to the HFD + SPSS group (Fig. 1L), suggesting that *L. plantarum* may benefit pancreatic and splenic injury in diabetic mice. These findings indicate that *L. plantarum* could counteract HFD-induced adverse effects and ameliorate pancreatic and liver damage in T2DM mice.

### Oral ingestion of *L. plantarum* modifies the gut microbiome composition in mice with T2DM

**Fig 2:**
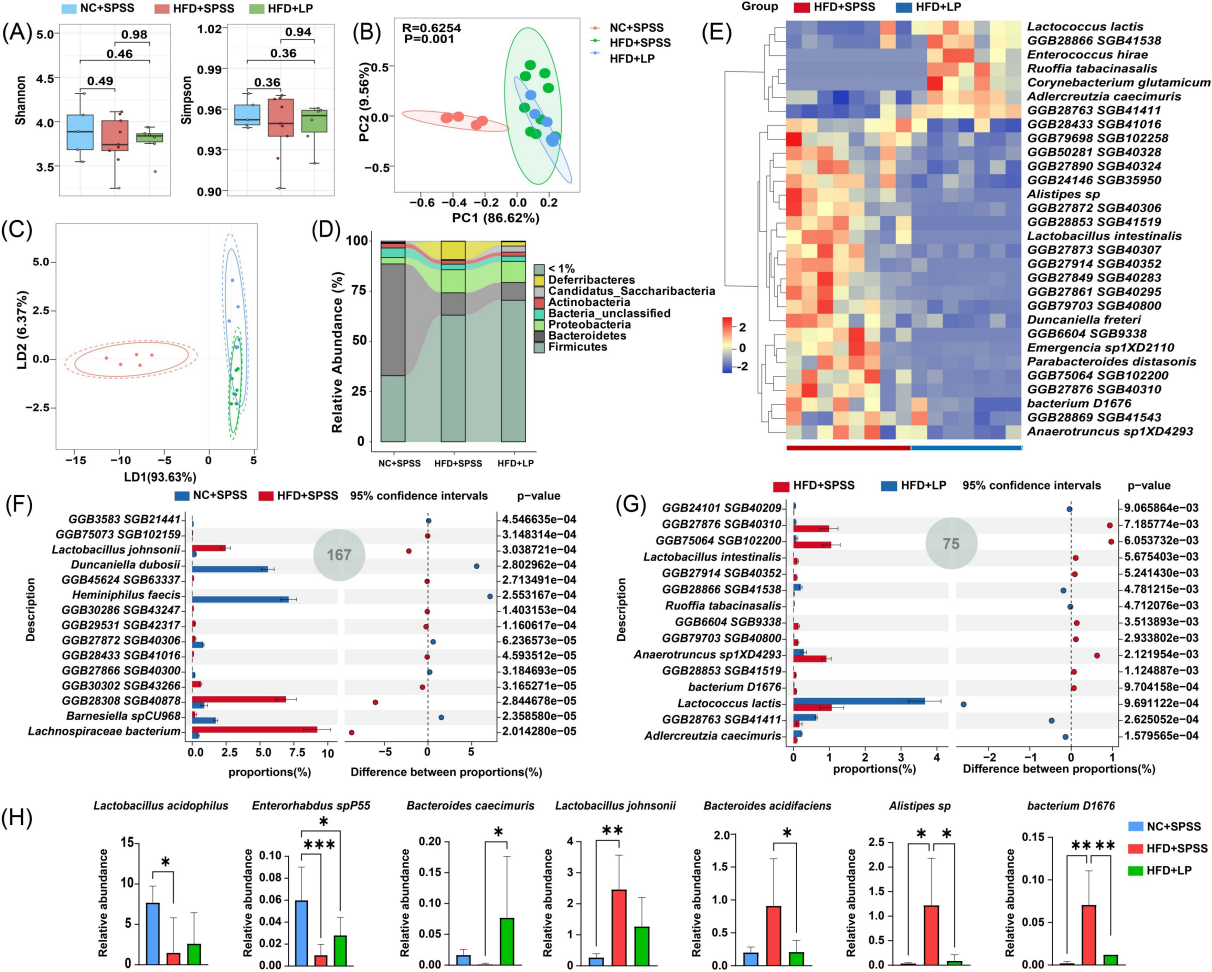
Significant alterations in gut microbiota taxonomic composition following oral administration of *L. plantarum* in T2DM mice. (**A**) Shannon and Simpson diversity indices for GM at the species level were compared. (**B**) Principal coordinate analysis of GM taxonomic composition. (**C**) Linear discriminant analysis (LDA) of gut microbiota taxonomic composition. (**D**) Histogram of species composition at phylum level. (**E**) The top 30 species were analyzed to reveal species-level alterations in GM composition. (**F**) The species-level GM within the NC + SPSS and HFD + SPSS groups was analyzed, with a 95% confidence interval. (**G**) The species-level GM composition in the HFD + LP and HFD + SPSS groups was investigated, with a 95% confidence interval. (**H**) Seven differential microbes of bacteria at species level. ****, *P* < 0.0001; ***, *P* < 0.001; **, *P* < 0.01; *, *P* < 0.05.These *P*-values denote statistical significance in comparison to the HFD + SPSS group.

To explore how probiotic supplementation influences the GM in mice with T2DM, we performed a comprehensive metagenomic analysis of microbial composition across all study groups. Comparative analysis of α-diversity indices (Shannon, Simpson) revealed no significant variations between HFD + SPSS and HFD + LP groups (*P* > 0.05, ANOVA; Fig. 2A). Further analysis of GM among all groups showed that *L. plantarum* supplementation led to a remodeling of GM in T2DM mice, suggesting a potential regulatory effect on the diabetic GM community (*P* < 0.001; Fig. 2B and C). The HFD + SPSS group exhibited a notable decrease in *Bacteroidota* compared to the NC + SPSS group. (11.14% ± 6.23%, *P* < 0.0001, *t* test; Fig. S2) and increases in *Firmicutes* (63.1% ± 9.14%, *P* < 0.0001, *t* test; Fig. S2B), *Deferribacteres* (9.09% ± 7.92%, *P* < 0.05, *t* test; Fig. S2), and *Proteobacteria* (11.48% ± 5.92%, *P* < 0.01, *t* test; Fig. S2D). In contrast, the HFD + LP group showed increased levels of *Firmicutes*, *Actinobacteria*, and *Candidatus Saccharibacteria* and decreased levels of *Bacteroidetes*, *Proteobacteria*, and *Deferribacteres* compared to the HFD + SPSS group (Fig. 2D).

Stamp analysis identified differential microbiota among different communities, with 167 differential microbes between the HFD + SPSS and NC + SPSS groups, and 75 between the HFD + SPSS and HFD + LP groups. Only the top 15 are shown in Fig. 2E. The HFD + SPSS group showed significant increases in *GGB28308 SGB40878* (6.92% ± 2.31%), *Lachnospiraceae bacterium* (9.22% ± 2.96%), and *Lactobacillus johnsonii* (2.47% ± 1.1%) and significant decreases in *Duncaniella dubosii* (0.01%) and *Heminiphilus faecis* (0.01%) compared with the NC + SPSS group. After the oral gavage of *L. plantarum*, *Lactococcus lactis* (3.67% ± 1.1%) levels rose in the GM of T2DM mice (*P* < 0.001, *t* test; Fig. 2F). Compared to HFD + SPSS, the HFD + LP group showed increased abundance of *L. acidophilus* (2.62% ± 3.83%) and *B. caecimuris* (0.08% ± 0.1%). Conversely, *Bacteroides acidifaciens* (0.21% ± 0.17%), *Alistipes* sp. (0.09% ± 0.12%), and *bacterium D1676* (0.01% ± 0.01%) were significantly reduced (*P* < 0.05, ANOVA; Fig. 2H). LEfSe analysis highlighted species-level microbiota alterations, identifying *L. johnsonii*, *B. acidifaciens*, *Alistipes* sp., and *bacterium D1676* as key taxa associated with *L. plantarum* intervention in T2DM mice (LDA >3.5, *P* < 0.05; Fig. S2E). Overall, these findings suggest that *L. plantarum* can alleviate obesity-related diabetes, with *L. lactis*, *L. acidophilus*, *Enterorhabdus* sp. P55 (*E.* sp. P55), and *B. caecimuris* potentially serving as key species contributing to its hypoglycemic effects.

### Identification of key gut metabolites following *L. plantarum* intervention

**Fig 3:**
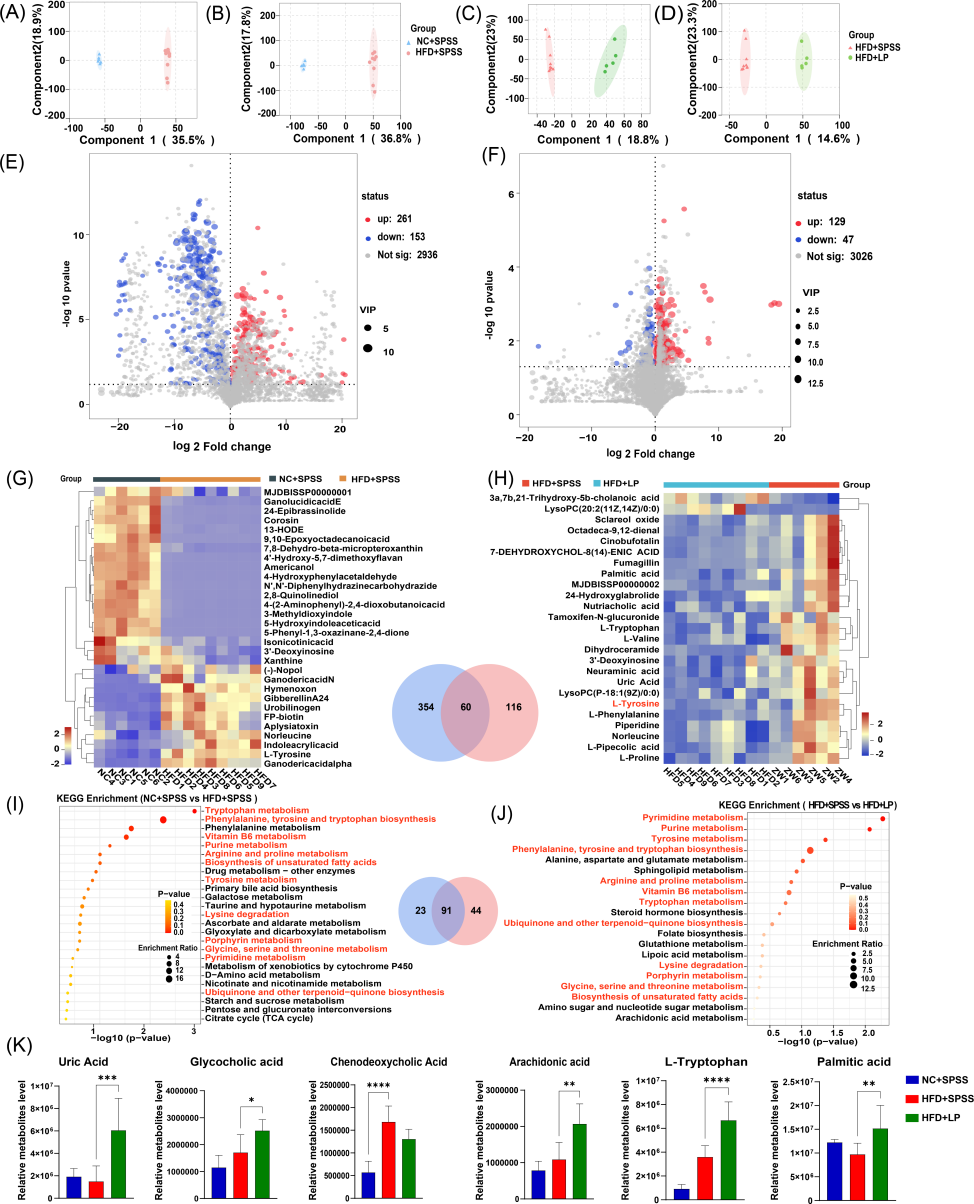
Identification of key gut differential metabolites and enriched pathways revealing the hypoglycemic mechanisms of *L. plantarum*. (**A, B**) OPLS-DA score plots for NC + SPSS and HFD + SPSS groups in ESI + and ESI− modes, respectively. (**C, D**) OPLS-DA score plots for HFD + SPSS and HFD + LP groups in ESI + and ESI− modes, respectively. (**E**) Volcanic map of DMs between the NC + SPSS and HFD + SPSS groups. (**F**) Volcanic map of DMs between HFD + SPSS and HFD + LP groups. (**G**) Top 25 DMs between NC + SPSS and HFD + SPSS groups. (**H**) Top 30 DMs between the HFD + SPSS and HFD + LP groups. Venn diagrams of all DMs between NC + SPSS and HFD + SPSS groups and HFD + SPSS and HFD + LP groups were visualized. KEGG pathway enrichment analysis for DMs in (**I**) NC + SPSS and HFD + SPSS groups and (**J**) HFD + SPSS and HFD + LP groups. Venn diagrams of all pathways between NC + SPSS and HFD + SPSS groups and HFD + SPSS and HFD + LP groups were visualized. (**K**) Effects of *L. plantarum* intervention on gut metabolites in T2DM mice. ****, *P* < 0.0001; ***, *P* < 0.001; **, *P* < 0.01; *, *P* < 0.05.These *P*-values denote statistical significance in comparison to the HFD + SPSS group.

Fecal samples from NC + SPSS (*n* = 6), HFD + SPSS (*n* = 9), and HFD + LP (*n* = 6) groups were analyzed by LC-MS. A total of 1,597 metabolites were identified via positive ion detection and 1,824 metabolites via negative ion detection. OPLS-DA score plots, which effectively illustrate model classification, revealed significant separation among all experimental groups (Fig. 3A through D). In the OPLS-DA model, metabolites with VIP >1.0 and *P* values <0.05 were identified as DMs.

Comparative analysis revealed 414 differential metabolites were found between the NC + SPSS and HFD + SPSS groups, with 153 upregulated [e.g., benzofenap, PI(PGE2/22:2(13Z,16Z)] and 261 downregulated [e.g., SM(d18:1/12:0), 12-hydroxydodecanoic acid] in the HFD + SPSS group (Fig. 3E). Meanwhile, 176 differential metabolites were identified between the HFD + SPSS and HFD + LP groups, with 129 upregulated (e.g., deoxycholylarginine, lysylglycine) and 47 downregulated [e.g., PE (15:0/14:1(9Z), apicidin] in the HFD + LP group (Fig. 3F). In the comparison between the NC + SPSS and HFD + SPSS groups, the top five contributors to the differences were 4-(2-aminophenyl)-2,4-dioxobutanoic acid, aplysiatoxin, americanol, norleucine, and 24-epibrassinolide (Fig. 3G). In the comparison between the HFD + SPSS and HFD + LP groups, the top five contributors to the differences were cinobufotalin, 7-dehydroxychol-8(14)-enic acid, 3'-deoxyinosine, lysoPC(P-18:1(9Z)/0:0), and neuraminic acid (Fig. 3H), with L-tyrosine shared between both comparisons. The KEGG pathway analysis of the 414 metabolites that varied significantly between the NC + SPSS and HFD + SPSS groups revealed a predominant role in the synthesis of amino acids such as phenylalanine, tyrosine and tryptophan biosynthesis, and tryptophan metabolism (Fig. 3I). KEGG enrichment analysis of the 176 DMs between HFD + SPSS and HFD + LP groups identified 135 pathways, indicating that *L. plantarum* treatment in diabetic mice primarily affected phenylalanine, tyrosine, and tryptophan, as well as pyrimidine metabolism (Fig. 3J). To verify *L. plantarum* impact on gut metabolites, the relative contents of six metabolites were measured. The HFD + SPSS group showed elevated glycocholic acid, chenodeoxycholic acid, and L-tryptophan levels but reduced uric acid and palmitic acid levels compared with the NC + SPSS group. *L. plantarum* treatment significantly reversed uric acid, chenodeoxycholic acid, and palmitic acid levels (*P* < 0.01, one-way ANOVA; Fig. 3K).

### *L. plantarum* attenuates HFD-induced T2DM symptoms through liver fatty acid metabolism and bile acid receptor activation

**Fig 4:**
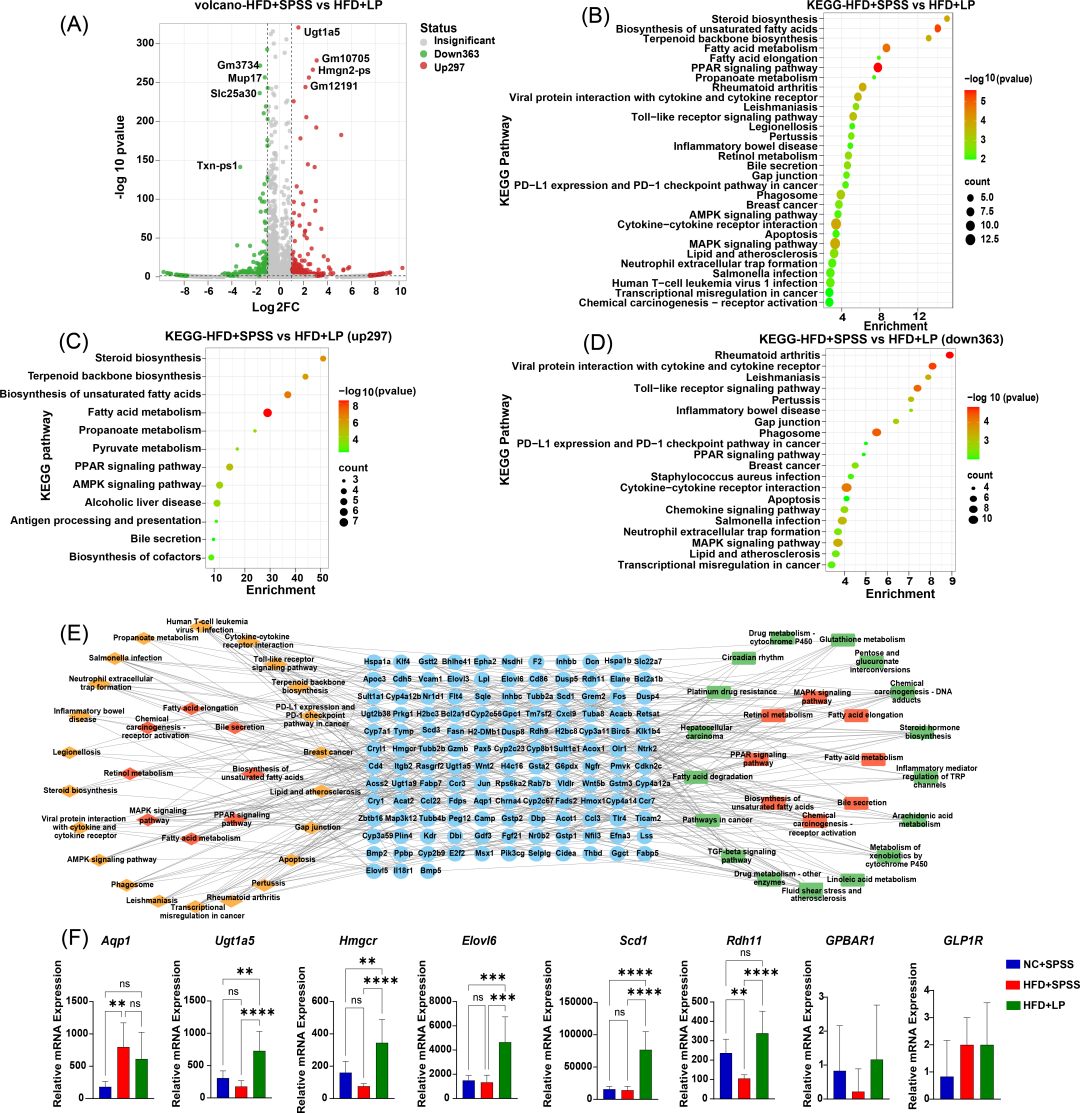
Transcriptomic analysis of *L. plantarum* intervention impact on liver gene expression in HFD-induced T2DM mice. (**A**) Volcano plot of DEGs in HFD + SPSS and HFD + LP Groups. (**B**) KEGG pathway enrichment analysis of all DEGs. (**C**) Upregulated DEGs were analyzed for pathway enrichment using the KEGG database. (**D**) Downregulated DEGs were subjected to KEGG pathway enrichment analysis. (**E**) The pathway-DEGs network of the HFD + SPSS vs HFD + LP and HFD + SPSS vs NC + SPSS. Yellow nodes represent pathways of the HFD + SPSS vs HFD + LP; red nodes represent common pathways; green nodes represent pathways of the HFD + SPSS vs NC + SPSS; and blue nodes represent common DEGs. (**F**) Effects of *L. plantarum* on related genes in liver of diabetic mice. ****, *P* < 0.0001; ***, *P* < 0.001; **, *P* < 0.01; *; *P* < 0.05.These *P*-values denote statistical significance in comparison to the HFD + SPSS group.

To investigate the impact of *L. plantarum* intervention on FBG reduction in HFD-induced mice, we conducted liver transcriptomic analysis across all experimental groups. Comparative analysis between the NC + SPSS and HFD + SPSS groups identified 1,486 differentially expressed genes (DEGs), including 1,201 upregulated genes (e.g., *Cyp2b9*, *Gm37795*, *Pitx3*, *Cidea*) and 285 downregulated genes (e.g., *Gm3543*, *Cyp2d32-ps*, *Gm32899*, *Gm2395*) (Fig. S3A). Notably, 660 DEGs (297 upregulated and 363 downregulated) were detected between the HFD + SPSS and HFD + LP groups. Prominently upregulated genes included *Ugt1a5*, *Gm10705*, *Hmgn2-ps*, and *Gm1219*, while *Gm3734*, *Mup17*, *Slc25a30*, and *Txn-ps1* exhibited significant downregulation (Fig. 4A).

KEGG enrichment analysis revealed distinct regulatory patterns. In the comparison between NC + SPSS and HFD + SPSS, DEGs were primarily enriched in cytokine–cytokine receptor interaction, phagosome, and leishmaniasis pathways (Fig. S3B). Upregulated genes showed significant enrichment in phagosome pathways and cytokine–cytokine receptor interaction (Fig. S3C), whereas downregulated genes were associated with chemical carcinogenesis–DNA adducts, drug metabolism–cytochrome P450 pathways, and retinol metabolism (Fig. S3D). In contrast, DEGs between HFD + SPSS and HFD + LP groups were mainly enriched in unsaturated fatty acid biosynthesis, PPAR signaling pathways, and fatty acid metabolism (Fig. 4B). Specifically, upregulated genes were functionally linked to fatty acid metabolism, unsaturated fatty acid biosynthesis, steroid biosynthesis, and terpenoid backbone biosynthesis (Fig. 4C). Conversely, downregulated genes were enriched in pathways related to rheumatoid arthritis, viral protein–cytokine receptor interaction, and Toll-like receptor signaling pathways (Fig. 4D).

We obtained potential DEGs associated with the NC + SPSS and HFD + SPSS groups, as well as between HFD + SPSS and HFD + LP groups. Enrichment analysis of these DEGs revealed their participation in shared pathways, including bile secretion, biosynthesis of unsaturated fatty acids, PPAR signal pathway, MAPK signaling pathway, fatty acid metabolism, retinol metabolism, chemical carcinogenesis–receptor activation, and fatty acid elongation. Subsequently, a pathway–gene network was constructed (Fig. 4E). A total of 135 genes were closely related to the 25 pathways in the comparison between the NC + SPSS and HFD + SPSS groups, and with 30 pathways in the comparison between the HFD + SPSS and HFD + LP groups. GO analysis results showed that, in biological processes (BP), DEGs were mainly correlated with negative regulation of Toll-like receptor 4 signaling pathway, lateral ventricle development, and terpenoid biosynthetic process. Regarding cellular components (CC), DEGs were predominantly associated with phagocytic cups and lipid droplets. Molecular function (MF) analysis was related to MAP kinase tyrosine phosphatase activity, protein tyrosine/threonine phosphatase activity, and MAP kinase tyrosine/serine/threonine phosphatase activity. These results suggest that *L. plantarum* may affect these pathways and attenuate obesity in mice (Fig. S3E).

To verify the impact of DEGs on the liver, we analyzed genes enriched in the bile acid pathway, fatty acid metabolism pathway, and retinal sulfonic acid pathway, including *Aqp1*, *Ugt1a5*, *Hmgcr*, *Elovl6*, *Scd1*, *Rdh11*, and G protein-coupled bile acid receptor1 (*GPBAR1/TRG5*) and *GLP1R* (Fig. 4F). Relative to the NC + SPSS group, the HFD + SPSS group exhibited upregulated *Aqp1* expression but downregulated *Ugt1a5*, *Hmgcr*, and *Rdh11* levels (*P* < 0.01, one-way ANOVA). *L. plantarum* treatment significantly reversed the expression levels of *Aqp1*, *Ugt1a5*, *Hmgcr*, *Elovl6*, *Scd1*, and *Rdh11* in T2DM mice (*P* < 0.01, one-way ANOVA). These results demonstrate that *L. plantarum* modulates the expression of bile acid-related genes in the liver.

### Integrated multi-omics analysis reveals mechanisms of *L. plantarum* hypoglycemic effects

#### Integrated analysis of microbiome and metabolome reveals GMs and DMs associations in T2DM

**Fig 5:**
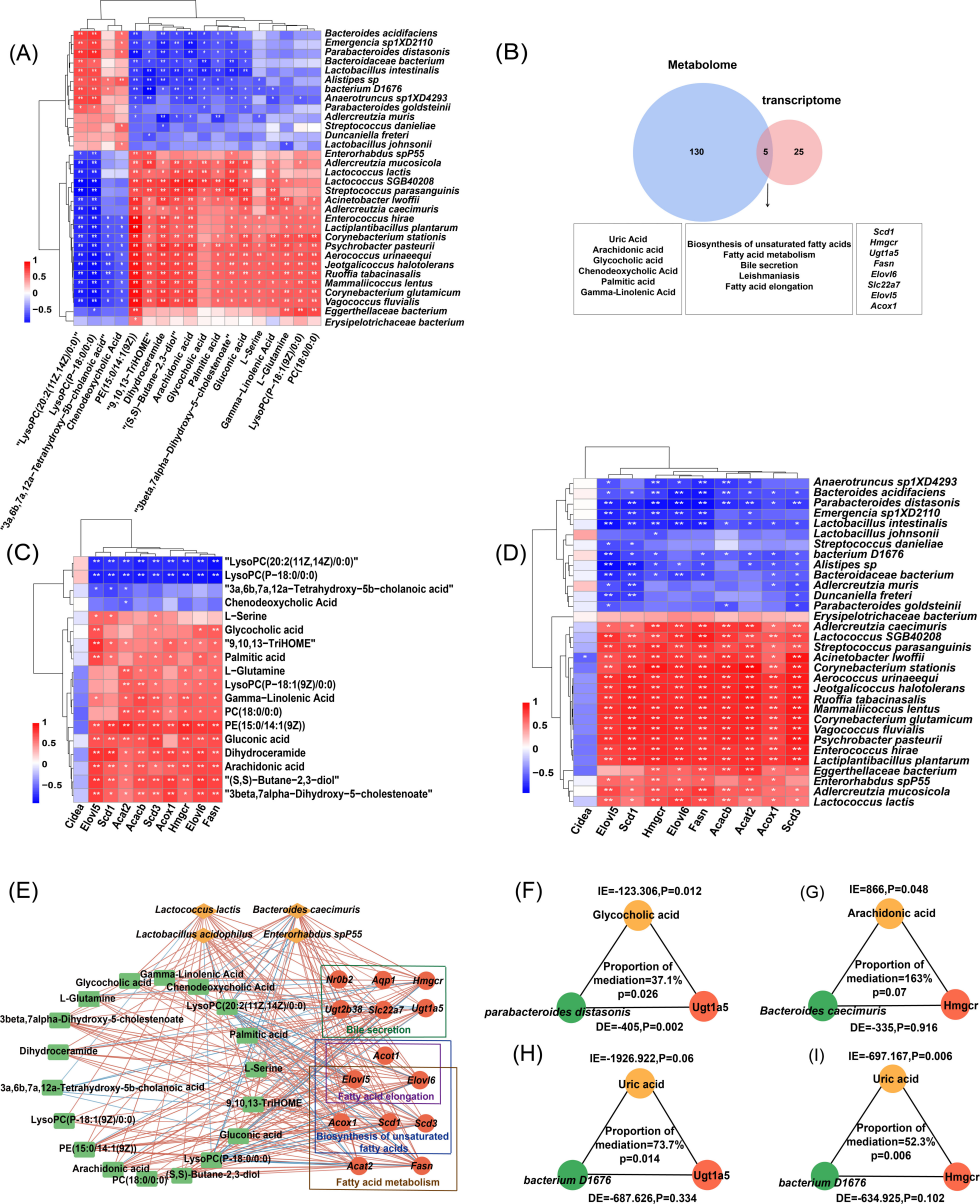
Multi-omics integration unlocks the mechanisms behind *L. plantarum* hypoglycemic effects. (**A**) Spearman correlations between GM at the species level and DMs. (**B**) Combined metabolome and transcriptome analysis. (**C**) Spearman correlation analysis between the metabolome and transcriptome. (**D**) Integrated microbiome and transcriptome analysis. (**E**) The key microbials are acting on the metabolites and genes in bile secretion, fatty acid metabolism, biosynthesis of unsaturated fatty acids, and fatty acid metabolism. Yellow nodes represent microbes; green nodes represent metabolites; and red nodes represent genes. Pathway affiliations are distinguished by colored borders surrounding each node. Red lines represent positive associations (*r* > 0.5), and blue lines indicate negative correlations (*r* < −0.5), with all displayed connections meeting statistical significance thresholds (*P* < 0.05). (**F–I**) *Parabacteroides distasonis* contributed to the upregulation of *Ugt1a5* by glycocholic acid (37.1%, Pmediation = 0.026) (**F**), and (**G**) arachidonic acid acted as a positive mediator linking *B. caecimuris* to the upregulation of *Hmgcr* (163% mediation, Pmediation = 0.07). Uric acid acted as a negative mediator linking *bacterium D1676* to the upregulation of (**H**) *Ugt1a5* (73.7% mediation, Pmediation = 0.014) and (**I**) *Hmgcr* (52.3% mediation, Pmediation = 0.006). IE (indirect effect) represents the causal pathway through the mediator. DE (direct effect) represents the unmediated regulatory impact of microbial species on gene expression.

Using Spearman’s correlation coefficient, we analyzed the correlations between gut microbiota composition and metabolic characteristics in samples from different groups. Integrative analysis revealed significant associations between 32 species-level microbial taxa and 18 metabolites (Fig. 5A). Spearman’s correlation at the species level was further visualized through a heatmap, delineating microbiota–metabolite interactions. Eighteen metabolites were selected that are involved in obesity-related pathways, including glycerophospholipid metabolism, bile acid biosynthesis, arachidonic acid metabolism, linoleic acid metabolism, and dicarboxylate metabolism. Among the metabolites engaged in these pathways, glycocholic acid showed strong positive associations with specific bacterial strains, including *Adlercreutzia mucosicola*, *L. lactis*, *Lactococcus* SGB40208, *Streptococcus parasanguinis*, *Acinetobacter lwoffii*, and *Psychrobacter pasteurii*. In contrast, it showed significant negative correlations with *Bacteroides acidifaciens*, *Emergencia* sp. 1XD2110, *Parabacteroides distasonis*, *Bacteroidaceae bacterium*, *Lactobacillus intestinalis*, *Alistipes* sp., *Bacterium* D1676, *Anaerotruncus* sp. 1XD4293, and *Parabacteroides goldsteinii*. Chenodeoxycholic acid exhibited significant positive correlations with *Bacteroides acidifaciens*, *Emergencia* sp. 1XD2110, *Parabacteroides distasonis*, *Alistipes* sp., *Streptococcus danieliae*, *L. johnsonii*, and negative correlations with *Enterococcus hirae*, *Lactiplantibacillus plantarum*, *Corynebacterium stationis*, *Psychrobacter pasteurii*, *Aerococcus urinaeequi*, *Jeotgalicoccus halotolerans*, *Ruoffia tabacinasalis*, *Mammaliicoccus lentus*, *Corynebacterium glutamicum*, and *Vagococcus fluvialis* (all *|r|* > 0.5, *P* < 0.05).

#### Integrated analysis of transcriptome and metabolome reveals DEGs and DMs associations in obesity pathways

Multi-omics integration of transcriptomic and metabolomic demonstrated coordinated alterations between DEGs and DMs. The results revealed changes in 135 KEGG metabolic pathways and 30 transcriptional pathways. Notably, five KEGG pathways showed overlapping changes in both transcriptomic and metabolomic. The metabolites involved in these five pathways include uric acid, arachidonic acid, glycocholic acid, chenodeoxycholic acid, palmitic acid, and gamma-linolenic acid. The genes involved in the five pathways include *Scd1*, *Hmgcr*, *Ugt1a5*, *Fasn*, *Elovl6*, *Slc22a7*, *Elovl5*, and *Acox1*. Among them, four pathways were related to obesity, namely, biosynthesis of unsaturated fatty acids, fatty acid metabolism, fatty acid elongation, and bile secretion (Fig. 5B). Among the genes involved in these pathways, glycocholic acid showed significant positive correlations with *Elovl5*, *Elovl6*, and *Fasn* (all *r* > 0.6, *P* < 0.01). Additionally, PE (15:0/14:1(9Z)), dihydroceramide, arachidonic acid, (S, S)-butane-2,3-diol, and 3β,7α-dihydroxy-5-cholestenoate were positively correlated with *Elovl5*, *Scd1*, *Acat2*, *Acacb*, *Scd3*, acyl-coenzyme A oxidase 1 (*Acox1)*, *Hmgcr*, *Elovl6*, and *Fasn* (all *r* > 0.5, *P* < 0.05, Fig. 5C). Combining transcriptomic and metabolomic data offers a holistic perspective on the molecular processes linked to obesity. The identified overlapping pathways highlight the importance of lipid metabolism and bile acid synthesis in obesity regulation.

#### Integrated microbiome and transcriptome profiling reveals host-microbe interactions in diabetic mice

We performed Spearman correlation analysis to assess the relationships between GM and DEGs aiming to elucidate the microbiota–liver crosstalk in obesity-related mechanisms. The analysis focused on several key pathways: biosynthesis of unsaturated fatty acids, bile secretion, fatty acid metabolism, and fatty acid elongation. These pathways play crucial roles in lipid metabolism and energy homeostasis, which are central to obesity pathogenesis. We identified several genes within these pathways that exhibited significant correlations with specific gut microbiota species. Notably, *Elovl5*, *Scd1*, *Hmgcr*, *Elovl6*, *Fasn*, *Acacb*, *Acat2*, *Acox1*, and *Scd3* showed positive correlations with 16 microbiota species, including *Adlercreutzia caecimuris Lactococcus SGB40208* (Spearman’s *r* > 0.5, *P* < 0.05; Fig. 5D). Conversely, these genes displayed negative correlations with *B. acidifaciens* and *Parabacteroides distasonis* (*r* < −0.5, *P* < 0.05)*.*

#### Integrated analysis of metagenome, transcriptome, and metabolome reveals key interactions in metabolic regulation

Network analysis integrating GM, metabolomic (18 DMs), and transcriptomic (14 DEGs) profiles identified key microbe–host interactions. Four microbial species exhibited significant correlations (Spearman, *P* < 0.05) with both metabolic shifts and gene expression changes, suggesting their potential involvement in systemic regulation (*P* < 0.05, Fig. 5E). Notably, intestinal bacteria including *L. lactis*, *L. acidophilus*, *B. caecimuris*, and *E.* sp. P55 interacted with host genes, participating in four key metabolic pathways, including bile secretion, fatty acid elongation, biosynthesis of unsaturated fatty acids, and fatty acid metabolism. Compared to the HFD + SPSS group, both HFD + LP and NC + SPSS groups showed significant upregulation of *Fasn*, *Acox1*, 3-hydroxy-3-methylglutaryl-coenzyme A reductase (*Hmgcr*), ELOVL family member 5 (*Elovl5*), stearoyl-coenzyme A desaturase 1 (*Scd1*), ELOVL family member 6 (*Elovl6*), and solute carrier family 22 (*Slc22a7*). Nuclear receptor subfamily 0 group B member 2 (*Nr0b2*) and UDP glucuronosyltransferase 2B38 (*Ugt2b38*) were significantly upregulated in the HFD + SPSS group compared to both the HFD + LP and NC + SPSS groups (*P* < 0.05). Mediation analyses identified glycocholic acid as a negative mediator linking *Parabacteroides distasonis* (37.1% mediation, Pmediation = 0.026) to the upregulation of *Ugt1a5* (Fig. 5F), and arachidonic acid as a positive mediator linking *B. caecimuris* to the upregulation of *Hmgcr* (163% mediation, Pmediation = 0.07, Fig. 5G). Uric acid was identified as a negative mediator linking *bacterium D1676* to the upregulation of *Ugt1a5* (73.7% mediation, Pmediation = 0.014) and *Hmgcr* (52.3% mediation, Pmediation = 0.006, Fig. 5H and I).

## DISCUSSION

In this study, we demonstrated the weight-reduction and glucose-lowering effects of *L. plantarum* through oral administration experiments in HFD-induced T2DM mice model. To elucidate its mechanistic basis, we employed an integrated approach to investigate tripartite interactions involving gut microbiota remodeling, microbial metabolite alterations, and liver gene expression regulation. Referring to our previous article (31), our findings revealed that *L. plantarum* supplementation significantly attenuated weight gain [ΔBW_(HFD+SPSS)_ = 4.71 g; ΔBW_(HFD+LP)_ = 1.7 g] and FBG levels [ΔFBG_(HFD+SPSS)_ = 1.54; ΔFBG_(HFD+LP)_ = −4.02] in diabetic mice, while concurrently enhancing glucose tolerance and insulin sensitivity.

Imbalances in the GM can disrupt the integrity of the intestinal barrier and influence host metabolism and signaling pathways, which are closely linked to T2DM (33). Earlier research has demonstrated that various *Lactobacillus* strains influence weight changes in a host-specific manner. For instance, certain *Lactobacillus* strains have been demonstrated to alleviate obesity or exhibit anti-obesity properties in HFD-fed mice (34). Conversely, other findings suggest that *L. fermentum*, in particular, may contribute to increased body weight (35). Compared to the HFD + SPSS group, the HFD + LP group exhibited significant increases in beneficial bacteria, including *L. acidophilus*, *L. lactis*, and *B. caecimuris*. This shift helped restore a healthier balance within the intestinal microbial community and suppressed the growth of harmful gut microbes. Our results revealed that increased in BW and FBG were positively correlated with the abundance of *Luteimonas* SGB9345, *Anaerotruncus* sp. 1XD42 93, and *Streptococcus acidominimus* (*P* < 0.01; Fig. S4B), but were negatively correlated with changes in *L. acidophilus* and *E.* sp. P55 (*r* < −0.7, *P* < 0.001; Fig. S4B through E). The genus *Enterorhabdus* has been reported by multiple studies as a bacterium associated with butyrate production (36, 37). Butyrate is a key short-chain fatty acid with multiple health benefits. It serves as the primary energy source for colonocytes and can improve metabolic health through anti-inflammatory and immunomodulatory effects (38, 39). Therefore, the significant correlation observed in this study between the abundance of *E.* sp. P55 and improved metabolic indicators suggest that it may exert protective effects in the host through the production of butyrate or similar beneficial metabolites. Notably, *L. acidophilus* is known for its ability to combat pathogenic bacteria, acting as a natural antagonist against potentially harmful microorganisms. This aligns with prior research findings (40). *Lactobacillus* showed positive correlations with three inflammatory factors, including significant positive correlations with IL-1β and TNF-α (41). These probiotics enhance gut barrier function, suppress inflammation, and improve insulin resistance by producing SCFAs through fermenting undigested carbohydrates.

Bile acids such as chenodeoxycholic acid (CDCA) and glycocholic acid, produced by the liver, aid fat digestion and nutrient absorption in the small intestine. Glycocholic acid often combines with taurine to influence bile secretion. Bile acids activate the TGR5 receptor, triggering glucagon-like peptide-1 (GLP-1) release, which enhances insulin secretion and pancreatic function. TGR5 plays a crucial role in glucose metabolism by promoting GLP-1 secretion from intestinal L cells. These findings emphasize that activating TGR5 via bile acids and the resulting GLP-1-mediated insulin control offer a promising strategy for treating T2DM (42–44). In our investigation, *L. plantarum* markedly elevated hepatic GLP-1 receptor levels in diabetic mice while upregulating TGR5 expression in the HFD + LP group supplemented with the probiotic. The observed shifts in bile acid profiles, along with concomitant changes in gene expression of TGR5, GLP-1R, and downstream targets, are consistent with potential modulation of the TGR5/GLP-1R signaling axis. Bile acids activate TGR5, influencing insulin secretion through the cAMP/PKA/CREB signaling cascade. Taurine-conjugated deoxycholic acid activates TGR5, enhancing insulin production. CREB controls proglucagon and PC1/3 gene expression, boosting GLP-1 secretion (45, 46). *L. plantarum* restored suppressed bile secretion in type 2 diabetic mice, indicating TGR5 activation as a key mechanism for enhanced GLP-1 secretion. It is noteworthy that these inferences are based on correlative evidence derived from metabolomic and transcriptomic data. Direct functional validation—such as measuring cAMP levels, GLP-1 secretion, or receptor phosphorylation—is required to definitively establish causal activation of this pathway. Our interpretation is strongly supported by specific alterations in the bile acid pool observed in our study, which align precisely with the well-established mechanism of TGR5/GLP-1R activation documented in the literature. For instance, the increase in secondary bile acids (such as DCA and LCA derivatives) we detected is highly significant, as these specific metabolites are well-characterized as potent natural agonists for TGR5 (47, 48). Their binding to TGR5 has been conclusively demonstrated to trigger intracellular cAMP accumulation (49) and subsequently stimulate GLP-1 secretion from enteroendocrine L cells (50). This cascade of events—from receptor binding to downstream hormone release and improved glucose homeostasis—has been firmly established through genetic gain- and loss-of-function studies (51).

Aquaporin-1 (*Aqp1*) enhances insulin sensitivity by maintaining cellular hydration and stability. This process boosts insulin-mediated glucose transporter 4 (GLUT4) translocation in adipocytes, thereby improving glycemic control. *Aqp1* also alters pro-inflammatory cytokine production and immune cell infiltration by changing macrophage polarization and reducing pro-inflammatory cytokine secretion (52). UDP-glucuronosyltransferase 1a5 (*Ugt1a5*), a key drug-metabolizing enzyme, has different expression levels in sexes and tissues. Its activity is crucial for xenobiotic clearance and pharmacokinetics, impacting the personalization of diabetes pharmacotherapy (53). HMG-CoA reductase (HMGCR) is a rate-limiting enzyme for cholesterol synthesis, and the regulation of its expression and activity is the key to regulating cholesterol synthesis. In addition, AMPK phosphorylates HMGCR (inactive form), and PP2A dephosphorylates phosphorylated HMGCR (active form) (54). AMPK is involved in the phosphorylation of S872 in the HMGCR catalytic domain (55), which leads to a decrease in the affinity of HMGCR for NADPH, thereby reducing enzyme activity and cholesterol synthesis through the mevalonate pathway. Notably, in this study, the AMPK signaling pathway in the liver was significantly activated, resulting in decreased *Hmgcr* activity and, consequently, reduced cholesterol synthesis. Transcriptome and metabolome profiling indicated the potential of *L. plantarum* to combat obesity through unsaturated fatty acid biosynthesis, bile production, and fatty acid metabolic pathways. Finally, it should be noted that the absence of a probiotic-intervention healthy control group prevents us from determining whether the observed effects are specific to the T2DM condition or represent a generalized response. Future studies comparing the responses of healthy cohorts and T2DM cohorts to *L. plantarum* supplementation will be essential to address this important question.

### Conclusion

**Fig 6:**
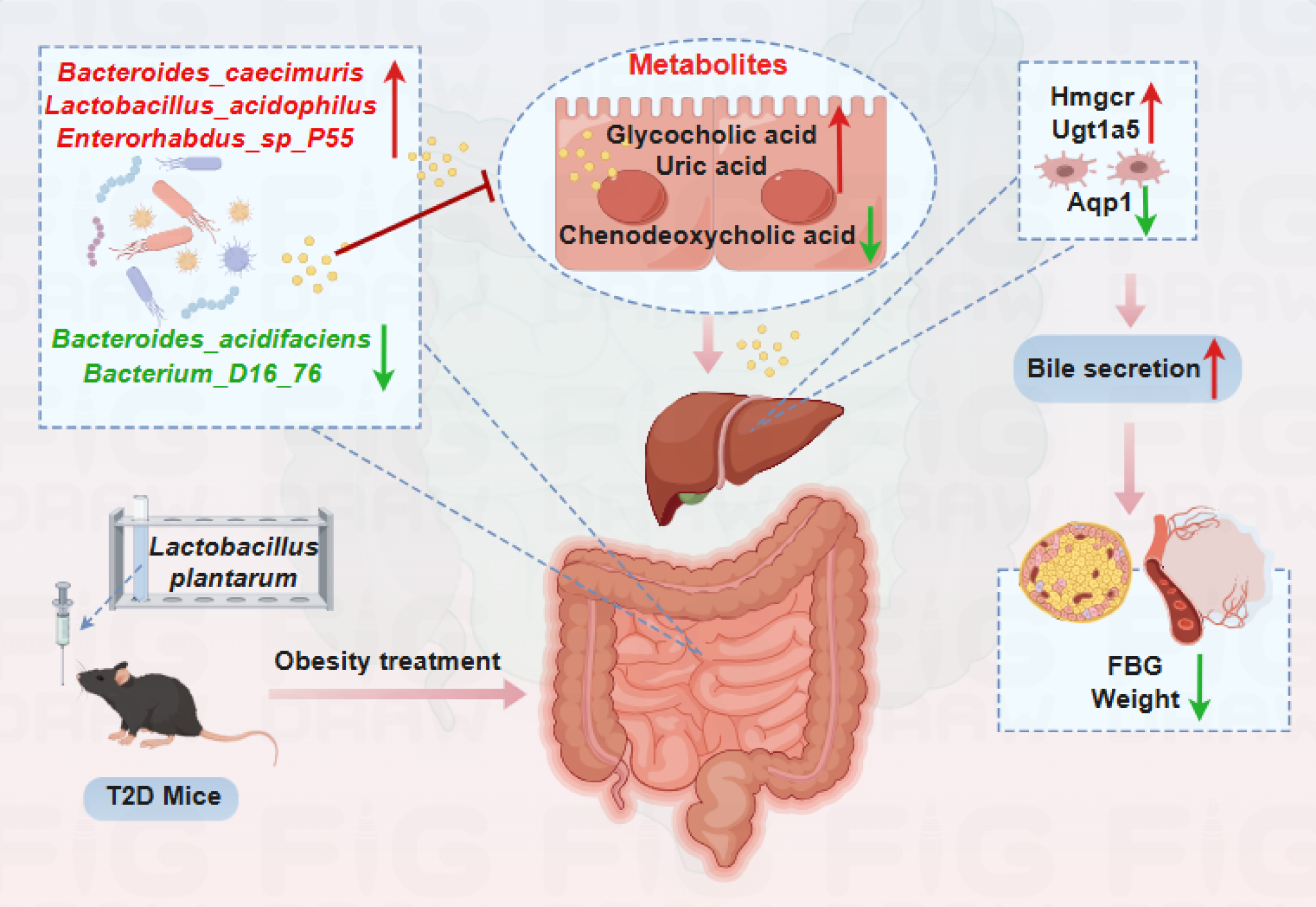
The mechanism by which *Lactobacillus plantarum* alleviates HFD-induced T2DM mice through the gut-liver axis.

T2DM has emerged as a worldwide health priority, urgently requiring the development of novel therapeutic interventions and elucidation of the underlying mechanisms. In this study, we employed *L. plantarum* intervention in animal models, combined with integrated multi-omics analysis, to elucidate the underlying mechanisms through which *L. plantarum* ameliorates T2DM via the gut-liver axis. In summary, *L. plantarum* can significantly reduce BW gain and FBG levels and protect liver and pancreatic tissue from damage in T2DM mice by regulating the gut microbiota, gut metabolites, and hepatic gene expression patterns (Fig. 6). *L. plantarum* achieved this by reducing the abundance of bacterium D1776, *B. acidifaciens*, and *P. distasonis*, while increasing the abundance of *L. acidophilus*, *E.* sp. P55*,* and *B. caecimuris*. These changes adjust bile secretion and its metabolites (including chenodeoxycholic acid and glycocholic acid), activate the expression of *TRG5* and *GLP-1R*, regulate blood glucose and lipid metabolic processes, enhance insulin production, and mitigate insulin resistance.

## Data Availability

Metagenomic sequencing data sets for fecal samples and transcriptome data for liver tissues in our study have been deposited into NCBI’s Sequence Read Archive (SRA) database with the BioProject number PRJNA1244596. Fecal metabolomic data sets were stored in the figshare database: DOI 10.6084/m9.figshare.28839806.

## SUPPLEMENTAL MATERIAL

Supplemental material msystems.00965-25-s0001.docx Supplemental text and figures.

ASM does not own the copyrights to Supplemental Material that may be linked to, or accessed through, an article. The authors have granted ASM a non-exclusive, world-wide license to publish the Supplemental Material files. Please contact the corresponding author directly for reuse.
