# Supplementary material for: Multi-omics analysis reveals the mechanism of Lactobacillus plantarum in alleviating metabolic disorders in type 2 diabetic mice through the gut-liver axis
Source: mSystems. 2025 Oct 30;10(11):e00965-25. doi: 10.1128/msystems.00965-25 (PMC12625740; doi:10.1128/msystems.00965-25)
Supplement: Supplemental material — Supplemental text and figures. [file msystems.00965-25-s0001.docx]

**Supplementary Material**

**Multi-omics analysis reveals the potential mechanisms of** ***Lactobacillus plantarum* alleviates metabolic disorders in type 2 diabetic mice through the gut-liver axis**

Caili Zhang^1, 2, †^, Yue Gao^1, 2, †^, Yixuan Huang^3, †^, Yunyi Qin^1, 2^, Yongxin Li^1, 2^, Qingru Chen^1, 2^, Tiantian Wu^1, 2^, Yujie Zhang^1, 2^, Yan Zhang^4^, Datong Deng^3^, Binbin Huang^5, *^, Mingwei Chen^3, *^, Maozhen Han^1, 2, *^

^1^ School of Life Sciences, Anhui Medical University, Hefei, Anhui 230032, China.

^2^ Microbial medicinal resources development research team, Anhui Provincial Institute of Translational Medicine, Hefei, Anhui 230032, China.

^3^ Department of Clinical Medicine, The First School of Clinical Medicine, Anhui Medical University, Hefei, Anhui, China.

^4^ School of Life Sciences, Hefei Normal University, Hefei, Anhui 230601, China.

^5^ School of Public Health, Anhui Medical University, Hefei, Anhui 230032, China.

†These authors contributed equally to this work.

^*^ Corresponding author E-mail: hanmz@ahmu.edu.cn; chmw1@163.com; huangbb91@126.com

**2. Materials and methods**

**2.3 Multi-omics analysis**

**2.3.1 Metagenome analysis**

The raw sequencing data underwent a standardized bioinformatics processing pipeline. Firstly, Raw sequencing data were quality-controlled and low-quality reads were filtered using fastp (v0.23.2) with default parameters. Secondly, to eliminate residual host-derived reads, the filtered reads were aligned with the reference genome (Mus musculus GRCm39) by using Bowtie2 (v2.4.5). The --un-conc parameter is used to output paired-end reads that fail to align to the host genome as separate paired-end files. Thirdly, taxonomic profiling was performed with MetaPhlAn4 (v4.0.6) and the default marker gene database (mpa_vJan21_CHOCOPhlAnSGB) was utilized.

**2.3.2 LC-MS analysis**

Fecal samples were collected and immediately frozen at -80°C for preservation. To extract metabolites, approximately 50 mg of feces were precisely weighed into 2 mL tubes, along with grinding beads and 400 µL of a methanol-water solution (4:1 ratio) spiked with internal standards. The samples were first homogenized cryogenically at -10°C for six minutes, then subjected to ultrasonic extraction for half an hour at 5°C (40 kHz). Following this, they were spun in a centrifuge at 13,000 g for 15 minutes while kept at -20°C. For optimal mass spectrometry performance, the instrument settings were fine-tuned with an electrospray voltage of 3.5 kV and the capillary heated to 325°C. Raw data were analyzed using Progenesis QI v3.0 software for peak alignment, metabolite identification, and MS/MS spectral matching. Only compounds with matching scores and mass accuracy errors below 10 ppm were considered for further analysis.

**2.3.3 Transcriptome analysis**

Liver tissue RNA was extracted using TRIzol reagent, and mRNA was purified via magnetic bead enrichment. The mRNA was fragmented to 200 base pairs, reverse transcribed into cDNA, and processed through ligation, PCR amplification, and purification to create the sequencing library. Library construction was done with the Illumina HiSeq X Reagent kit (Illumina, San Diego, USA), and sequencing was performed using the DNBSEQ-T7RS Reagent Kit. After removing the connector sequence and low-quality reads using fastp (v0.23.2) with default parameters, the raw reads were converted into clean reads. These clean reads were then aligned to the mouse reference genome (GRCm38) using the HISAT2 software (v2.2.1). Subsequently, the subread software (v2.0.2) was employed to accurately count the reads. Finally, all sample read counts were combined into one file to determine each gene's expression level across samples. Gene expression matrices were analyzed using DEseq2 (v1.42.1). DEGs were defined with thresholds of |log2Fold Change| > 1 and adjusted *p*-value (FDR) <0.05, and adjusted *p*-value were obtained using the Benjamini / Hochberg method. Subsequently, functional enrichment analysis was conducted using the Kyoto Encyclopedia of Genes and Genomes (KEGG) database, while Gene Ontology (GO) pathway analysis was performed via the Metascape platform. (1).

**2.4 Statistical analysis**

GraphPad Prism (v9.5.1) was used for statistical analyses, all data were presented as “mean ± standard error mean (SEM)”, and p < 0.05 was considered statistically significant (**p* < 0.05; ***p* < 0.01; ****p* < 0.001; *****p* < 0.0001). Two-tailed Student’s t-test (for parametric data) or a Mann-Whitney U test (for non-parametric data) was used to evaluate the significance of differences between the two groups. When dealing with more than two groups, a one-way ANOVA was used to gauge significance, followed by either Tukey’s or Dunnett’s post hoc tests (for parametric data) or the Kruskal-Wallis test with Dunn’s method for multiple comparisons (for non-parametric data). The "anosim" function from the "vegan" package was used to compute p-values and r-values. Principal Coordinates Analysis (PCoA) was performed using the “stats” package (v3.5.0) and visualized with “ggplot2” (v3.2.0). Linear discriminant analysis (LDA) was executed with the lda function from the “MASS” package (v7.3-60). Heatmap was performed using the “ComplexHeatmap” package (v2.12.0). Mediation analysis was performed using the “mediation” package (v4.5.0) and “plyr” package (v1.8.9). Additionally, cytoscape (v3.9.1) was employed for graphically representing the intercorrelations among different bacterial species.

**Supplementary figures**

**Figure. S1.** The results of the phylogenetic tree construction

**Figure. S2.** The results of phylum-level differences between normal control mice and HFD - induced T2DM mice, and the biological markers identified by LEfSe, visually show changes in gut microbiota bacterial classification at the species level.

**Figure. S3.** Transcriptomic analysis of the effects of liver gene expression in normal controls and HFD-induced T2DM mice.


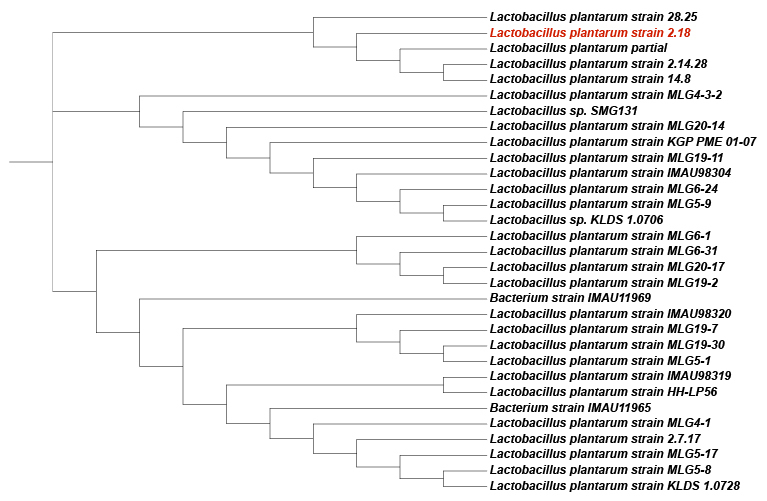


**Figure. S1. The results of the phylogenetic tree construction.** The phylogenetic tree results show that this bacterium belongs to *Lactobacillus plantarum*.


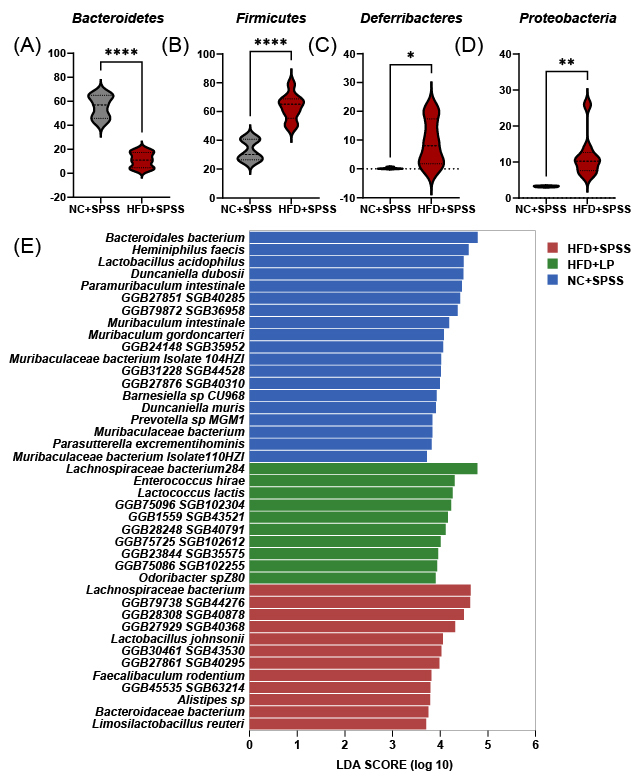


**Figure. S2. (A-D)** the results of phylum-level differences between normal control mice and HFD - induced T2DM mice, and **(E)** the biological markers identified by LEfSe, visually show changes in gut microbiota bacterial classification at the species level.


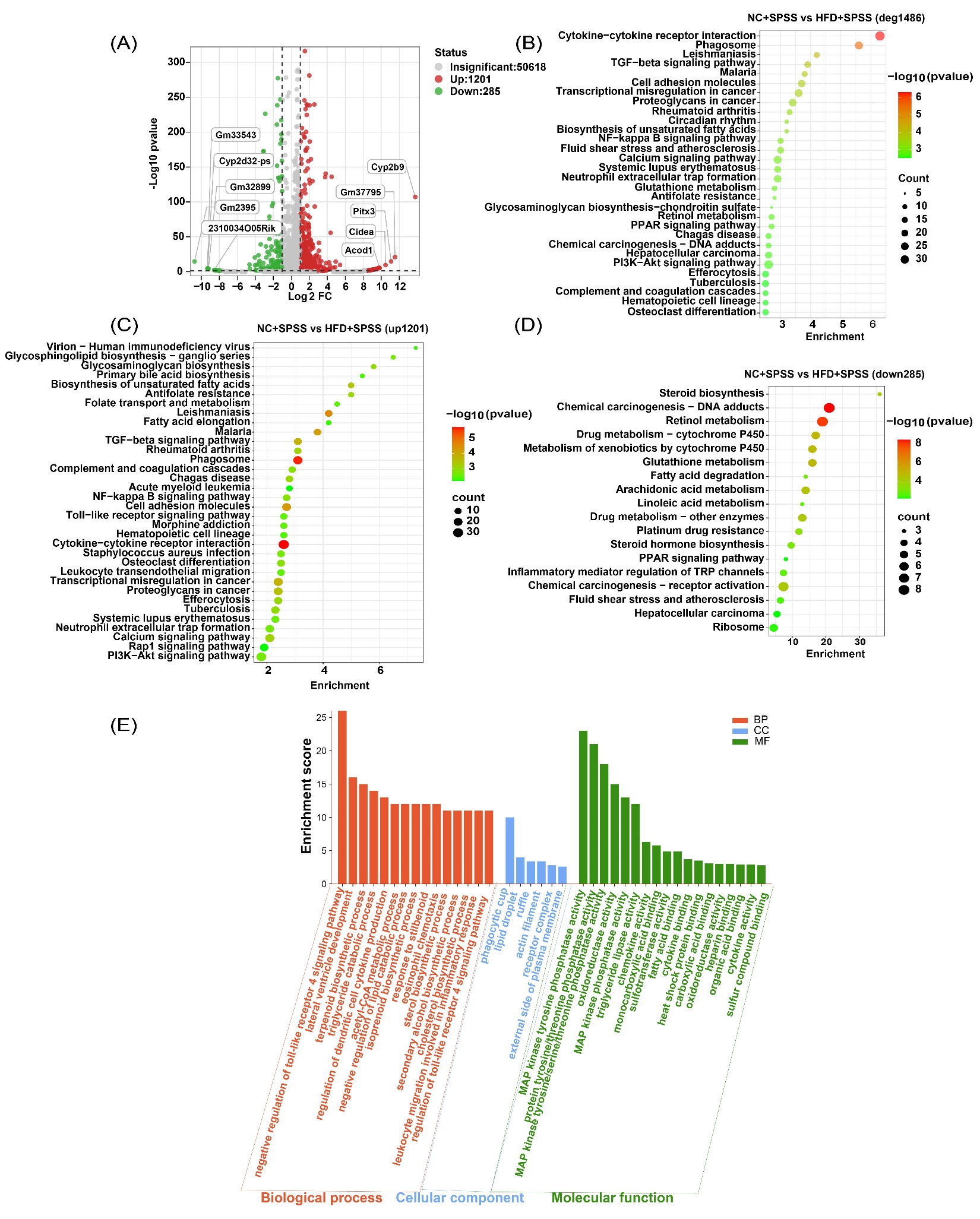


**Figure. S3. Transcriptomic analysis of the effects of liver gene expression in normal controls and HFD-induced T2DM mice.** **(A)** Volcanic map of differentially expressed genes. **(B)** KEGG pathway enrichment analysis of down-regulated genes. **(C)** KEGG pathway enrichment analysis of up-regulated gene. **(D)** KEGG pathway enrichment analysis of DEGs. **(E)** GO pathway enrichment analysis of DEGs


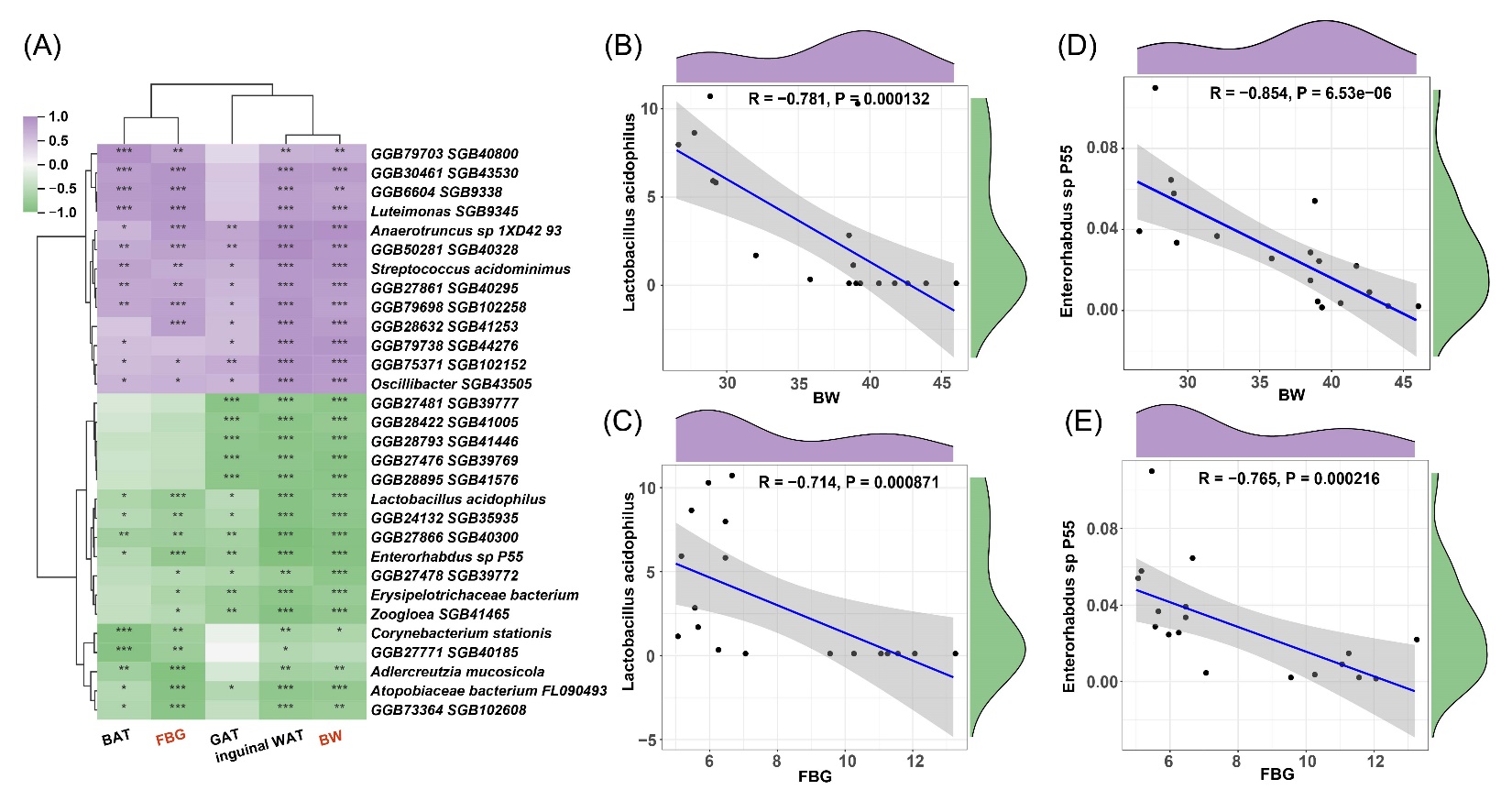


**Figure. S4.** Association between phenotypic factors and microbiota analyzed by Spearman correlation. (**A**) Heatmap showing the correlations between physiological parameters (BAT, FBG, GAT, inguinal WAT, BW) and gut microbiota. (**B, C**) Scatter plots demonstrating the significant negative correlations of *Lactobacillus acidophilus* with BW and FBG (*r* < -0.7, *p* < 0.001). (**D, E**) Scatter plots demonstrating the significant negative correlations of *Enterorhabdus sp. P55* with BW and FBG (*r* < -0.7, *p* < 0.001)

1. Zhao X, Zhang L, Wang J, Zhang M, Song Z, Ni B, You Y. 2021. Identification of key biomarkers and immune infiltration in systemic lupus erythematosus by integrated bioinformatics analysis. J Transl Med 19:35.
